# A novel silkworm infection model with fluorescence imaging using transgenic *Trichosporon asahii* expressing eGFP

**DOI:** 10.1038/s41598-020-67841-6

**Published:** 2020-07-03

**Authors:** Yasuhiko Matsumoto, Hideki Yamazaki, Yusuke Yamasaki, Yuki Tateyama, Tsuyoshi Yamada, Takashi Sugita

**Affiliations:** 1 Department of Microbiology, Meiji Pharmaceutical University, 2-522-1, Noshio, Kiyose, Tokyo 204-8588 Japan; 2 Teikyo University Institute of Medical Mycology, 359 Otsuka, Hachioji, Tokyo 192-0395 Japan; 3 Asia International Institute of Infectious Disease Control, Teikyo University, 2-11-1, Kaga, Itabashi-ku, Tokyo, 173-8605 Japan

**Keywords:** Animal disease models, Small molecules, Fungal infection

## Abstract

*Trichosporon asahii* is a pathogenic fungus that causes deep mycosis in patients with neutropenia. Establishing an experimental animal model for quantitatively evaluating pathogenicity and developing a genetic recombination technology will help to elucidate the infection mechanism of *T. asahii* and promote the development of antifungal drugs. Here we established a silkworm infection model with a transgenic *T. asahii* strain expressing eGFP. Injecting *T. asahii* into silkworms eventually killed the silkworms. Moreover, the administration of antifungal agents, such as amphotericin B, fluconazole, and voriconazole, prolonged the survival time of silkworms infected with *T. asahii*. A transgenic *T. asahii* strain expressing eGFP was obtained using a gene recombination method with *Agrobacterium tumefaciens*. The *T. asahii* strain expressing eGFP showed hyphal formation in the silkworm hemolymph. Both hyphal growth and the inhibition of hyphal growth by the administration of antifungal agents were quantitatively estimated by monitoring fluorescence. Our findings suggest that a silkworm infection model using *T. asahii* expressing eGFP is useful for evaluating both the pathogenicity of *T. asahii* and the efficacy of antifungal drugs.

## Introduction

*Trichosporon asahii* is a basidiomycetous yeast widely distributed in the environment^1–4^, and commonly isolated from human blood, sputum, skin, feces, and urine^5–8^. *T. asahii* is a pathogenic fungus that causes severe deep mycosis in patients with neutropenia^8–10^. Whereas the mortality rate of deep mycosis caused by *Candida albicans* is approximately 40%, that caused by *T. asahii* is approximately 80%, and the prognosis is poor^11^. *T. asahii* is resistant to echinocandin antifungal drugs and causes severe infections in patients treated with micafungin^12^. Moreover, strains resistant to amphotericin B and azole antifungal drugs such as fluconazole have been isolated from patients^13,14^. Therefore, *T. asahii* has become a serious clinical problem as a pathogenic fungus that causes systemic infection in immunocompromised hosts^8^.

*T. asahii* forms hyphae, which are branching filamentous structures^14^. In pathogenic fungi that form hyphae such as *Candida albicans*, hyphal formation is crucial for the host epithelial cell damage and biofilm formation that are involved in infections^15^. As with *C. albicans*, *T. asahii* makes treatment difficult by forming a biofilm on devices such as catheters^14,16^. Hyphal growth of *T. asahii* in blood vessels causes necrotic thrombi, and may contribute to infection^17^. The molecular mechanisms of infection caused by *T. asahii*, however, remain unclear. Establishing a simple animal experimental system for systemic infection with hyphal formation of *T. asahii* and developing genetic recombination technology in *T. asahii* will contribute to elucidate the infection mechanism.

The silkworm, an invertebrate, is a useful experimental animal for evaluating the pathogenicity of pathogenic microorganisms and the therapeutic effects of antibacterial, antifungal, and antiviral drugs^18–22^. Silkworms have advantages for conducting infection experiments that require a large number of individuals^23–25^ as they are much less expensive to rear and maintain than mammals and their experimental use partly avoids the ethical problems associated with that of mammalian models^23,24^. These advantages allow for the use of a large number of silkworms to calculate the dose of a pathogen required to kill half of the silkworms (LD_50_) and the administration dose of an antifungal drug required to promote survival in half of the silkworms (ED_50_) for quantitative evaluation of the pathogenicity of microorganisms and the efficacy of therapeutic agents^26–28^. Furthermore, silkworm infection models can be used for in vivo screening experiments, and allow for the identification of virulence-related genes of microorganisms and therapeutically effective drugs. Virulence-related genes of pathogenic microorganisms such as *Staphylococcus aureus*, *Candida albicans*, and *Candida glabrata* have been discovered using silkworm infection models and gene-deficient mutant libraries^19,29,30^. Avirulent mutants that exhibit lower pathogenicity against silkworms also exhibit lower pathogenicity in infection experiments in mice. Many studies using antimicrobial drugs, toxic compounds, and natural products demonstrate that the therapeutic effects, pharmacokinetic parameters, and toxicity are similar between silkworms and mammals^31–35^. Moreover, lysocin E^36^, nosokomycin^37^, ASP2397^38^, and GPI0363^34^ were identified by exploratory research using a silkworm infection model from microbial culture broths and chemical libraries as novel antimicrobial compounds that show therapeutic effects in mouse infection experiments. Therefore, the silkworm infection model is useful for elucidating pathogenic mechanisms and evaluating the efficacy of antifungal drugs^24,39^.

Fungal hyphae comprise multiple cells, making it difficult to quantitatively estimate hyphal formation by counting colony-forming units^40^. Fluorescence imaging using enhanced green fluorescent protein (eGFP) is a simple and effective method for quantitatively evaluating cell proliferation and cell death in vivo^41,42^. We previously established a silkworm infection model using transgenic dermatophytes expressing eGFP, and developed a method for quantifying hyphal formation by fluorescence imaging^43^.

In the present study, we demonstrated that *T. asahii* can kill silkworms and successfully established a transgenic *T. asahii* strain expressing eGFP by genetic recombination using *Agrobacterium tumefaciens*. The therapeutic effects of antifungal drugs can be evaluated using a silkworm infection model with the transgenic *T. asahii* strain expressing eGFP. The silkworm infection model and gene recombination technology established in this study were effective toward elucidating the infection mechanism of *T. asahii*.

## Material and methods

### Culture of *T. asahii*

*T. asahii* JCM2466 was grown on Sabouraud dextrose agar plates and incubated at 27 °C for 2 days.

### Silkworm rearing

Eggs of silkworms were purchased from Ehime-Sanshu Co., Ltd. (Ehime, Japan), disinfected, and hatched at 25–27 °C. The silkworms were fed an artificial diet, Silkmate 2S, containing antibiotics purchased from Ehime-Sanshu Co., Ltd. (Ehime, Japan). Fifth instar larvae were used in the infection experiments.

### Silkworm infection experiments

Silkworm fifth instar larvae were fed an artificial diet (Silkmate 2S; Ehime-Sanshu Co., Ltd., Ehime, Japan) overnight. *T. asahii* grown on Sabouraud agar plates was suspended in physiological saline solution (0.9% w/v NaCl) and filtered through a 40-µm cell strainer (Corning, NY, USA). A suspension (50 μl) of the *T. asahii* cells was injected into the silkworm hemolymph using a 1-ml tuberculin syringe (Terumo Medical Corporation, Tokyo, Japan). Silkworms injected with *T. asahii* cells were placed in an incubator and their survival was monitored.

### LD_50_ measurement

*T. asahii* JCM2466 (2.9 × 10^3^ to 1.8 × 10^7^ cells) was injected into the silkworm hemolymph. Survival of the silkworms at 48 h was monitored. The LD_50_, which is the dose of *T. asahii* required to kill half of the silkworms, was determined from combined data of 6 independent experiments by simple logistic regression model using Prism 8 (GraphPad Software, LLC, San Diego, CA, USA, https://www.graphpad.com/scientific-software/prism/).

### Antifungal agents

Amphotericin B (Wako, Osaka, Japan), fluconazole (Wako, Osaka, Japan), and voriconazole (Tocris Bioscience, Bristol, UK) were dissolved in dimethyl sulfoxide (Wako, Osaka, Japan) and stored at − 80 °C until use.

### Minimum inhibitory concentration determination

The minimum inhibitory concentration (MIC) was determined using a drug sensitivity test kit, yeast-like fungi DP (Eiken Chemical, Tokyo, Japan), according to the Clinical and Laboratory Standards Institute (CLSI) M27-A3 method. Briefly, *T. asahii* cells (2 × 10^3^ cells per well of a 96 well plate) were incubated with twofold serial dilutions of antifungal agents at 37 °C for 2 days, and the MIC values were determined.

### ED_50_ measurement

To evaluate the therapeutic effects of antifungal agents, *T. asahii* cells (1–5 × 10^6^ cells) were injected into the silkworm hemolymph, and various concentrations of the antifungal agents (50 μl) dissolved in saline were injected immediately afterwards into the silkworm hemolymph. The doses were created by fourfold serial dilutions. To determine the ED_50_ values, five or six silkworms were injected with each dose of the antifungal agents. Survival of the silkworms at 48 h was monitored. The ED_50_ values were calculated from combined data of 4–5 independent experiments by simple logistic regression model using Prism 8 (GraphPad Software, LLC, San Diego, CA, USA, https://www.graphpad.com/scientific-software/prism/).

### Construction of *T. asahii* expressing eGFP

The plasmid for expressing eGFP in *T. asahii* was constructed according to a previous report^43^. The eGFP gene was introduced into a pAg1-NAT1 vector^44^. The promoter sequence of the actin gene of *Cryptococcus neoformans* (CnPactin) was incorporated upstream of the eGFP gene, and the termination sequence of the *Aspergillus nidulans trpC* gene was incorporated downstream of the eGFP gene^45^. Cloning was performed using the infusion method according to the general method (In-Fusion HD Cloning Kit, Takara, Shiga, Japan). The primers used for polymerase chain reaction (PCR) amplification of each DNA region are shown in Table 1.

**Table 1:** Primers used in the study.

Primers	Nucleic acid sequence
**pAg1-NAT for infusion cloning**	
FpAg1-NAT	AGGATCCTTGCGCGCCTAGGC
RpAg1-NAT	GAAGAGATGTAGAAACTAGCT
**CnPactin for infusion cloning**	
FCnPactin(-pAg1-NAT)	TTTCTACATCTCTTCGCTGCGAGGATGTGAGCTGGA
RCnPactin(-eGFP)	CTCGCCCTTGCTCACCATGCCTCGATGGCCTCGGCGTCC
**eGFPTtrpC for infusion cloning**	
FeGFPTtrpC(-CnPactin)	TTTCTACATCTCTTCATGGTGAGCAAGGGCGAGGAG
ReGFPTtrpC(-pAg1-NAT)	GCGCGCAAGGATCCTAAAGAAGGATTACCTCTAAACAAGTGTACC

The pAg1-NAT1-eGFP was introduced into the *T. asahii* JCM2466 strain using the *A. tumefaciens*-mediated transformation method described previously^46,47^. The pAg1-NAT-eGFP was introduced into *A. tumefaciens* EHA105 strain by electroporation and transformants were grown on 2 × YT agar containing rifampicin (50 µg/ml), chloramphenicol (25 µg/ml), and kanamycin (50 µg/ml). The transformant was co-cultured with the *T. asahii* JCM2466 strain at 27 °C for 2 days. The candidate transgenic *T. asahii* expressing eGFP were isolated as colonies grown on Sabouraud dextrose agar containing nourseothricin (50 µg/ml) and cefotaxime (100 µg/ml). Introduction of the eGFP gene into genome of the candidate strains was confirmed by PCR using the primers described in Table 1.

### Imaging of eGFP-expressing *T. asahii* in silkworms

Fluorescence imaging using eGFP-expressing fungi in silkworms was performed according to a previous report^43^. eGFP-expressing *T. asahii* cells (2 x 10^6^ cells) were injected into silkworms. The silkworms were reared at 37 °C and their hemolymph was collected after 24 h. The hemolymph was placed on glass slides and covered by a glass coverslip. The samples were examined with bright light or ultraviolet light under a microscope equipped with a fluorescence lens (DP-74; Olympus, Tokyo, Japan). The pictures were randomly obtained and the image fluorescence was determined by Image J software (ImageJ 1.47t; National Institutes of Health, Bethesda, MD, https://imagej.nih.gov/ij/).

### Statistical analysis

The significance of differences between groups was calculated using the Tukey–Kramer method. *P* < 0.05 was considered significant.

## Results

### Killing of silkworms by injection of *Trichosporon asahii* cells

**Figure 1:**
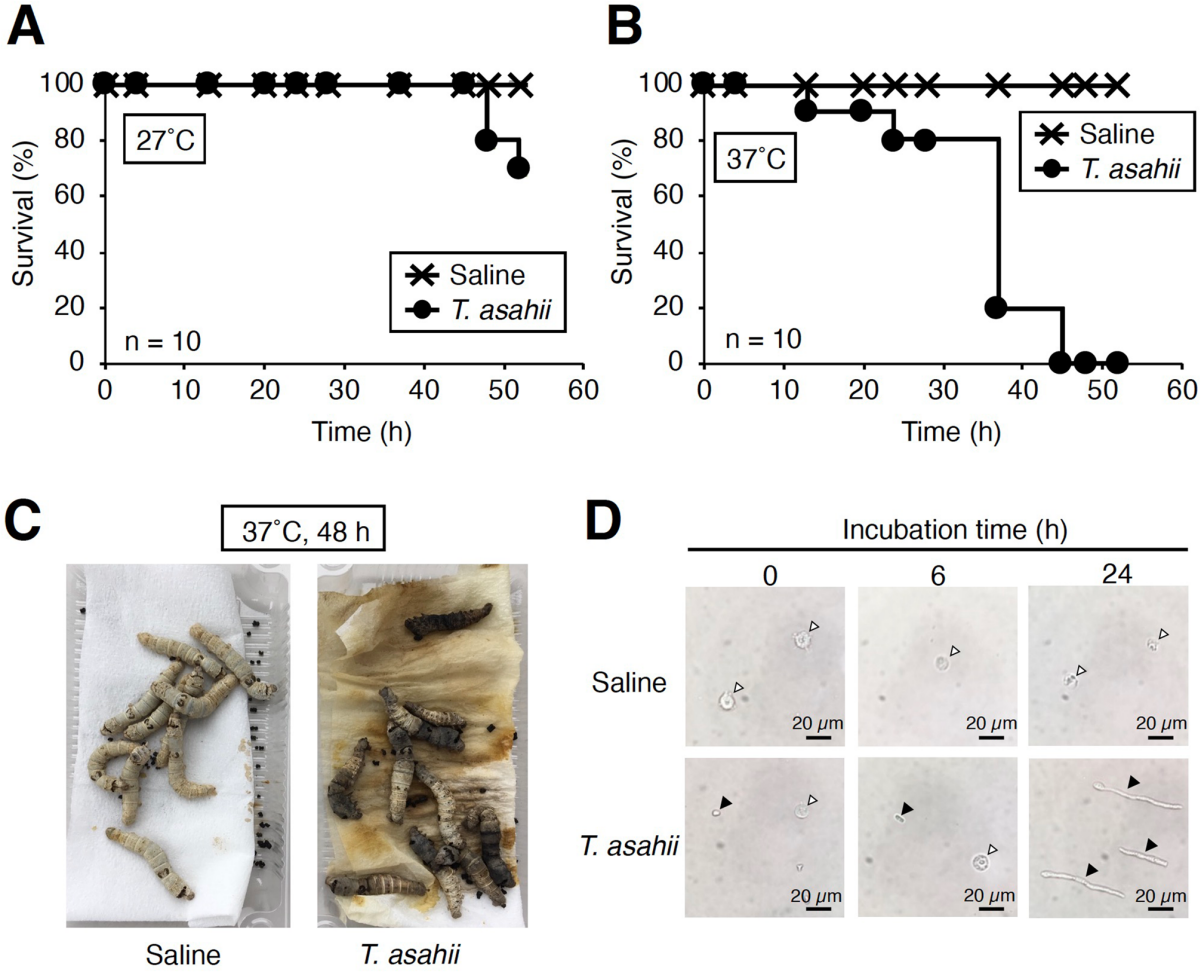
Killing of silkworms by injection of *T. asahii*. Saline or *T. asahii* JCM2466 (2 × 10^6^ cells) was injected into the silkworm hemolymph. Survival of the silkworms at 27 °C (**A**) and 37 °C (**B**) was monitored. n = 10/group. (**C**) Silkworms after injection at 48 h under 37 °C incubation are shown. (**D**) Saline or *T. asahii* JCM2466 (2 × 10^6^ cells) was injected into the silkworm hemolymph. Silkworm hemolymph was collected at 0, 6, and 24 h after injection and observed with a microscope. White arrow indicates hemocytes of silkworm. Black arrowheads indicate *T. asahii* cells.

**Figure 2:**
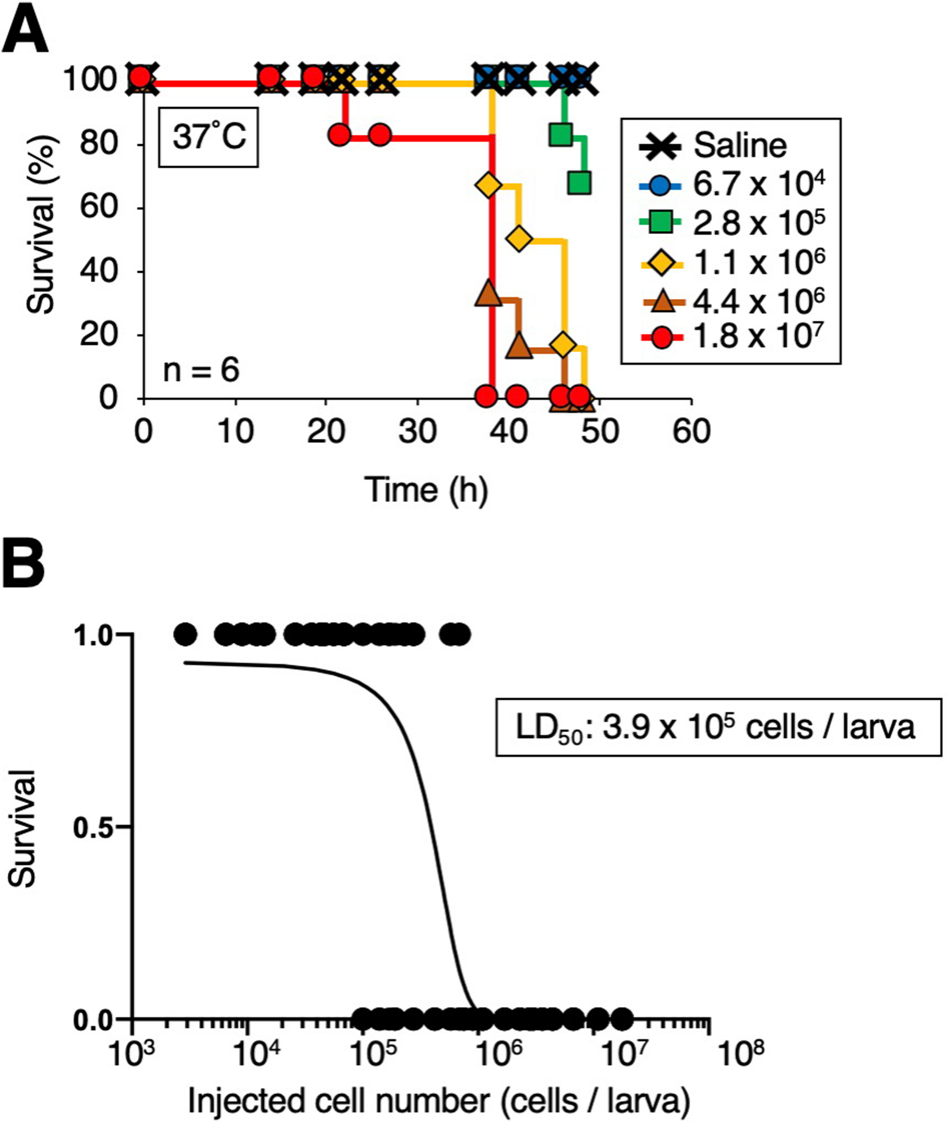
Death of silkworms according to the number of *T. asahii* cells administered. (**A**) Saline or *T. asahii* JCM2466 (6.7 × 10^4^ – 1.8 × 10^7^ cells) was injected into the silkworm hemolymph. Survival of the silkworms at 37 °C was monitored. n = 6/group. (**B**) Survival of the silkworms at 48 h was monitored. The LD_50_, which is the dose of *T. asahii* required to kill half of the silkworms, was determined from combined data of six independent experiments.

**Figure 3:**
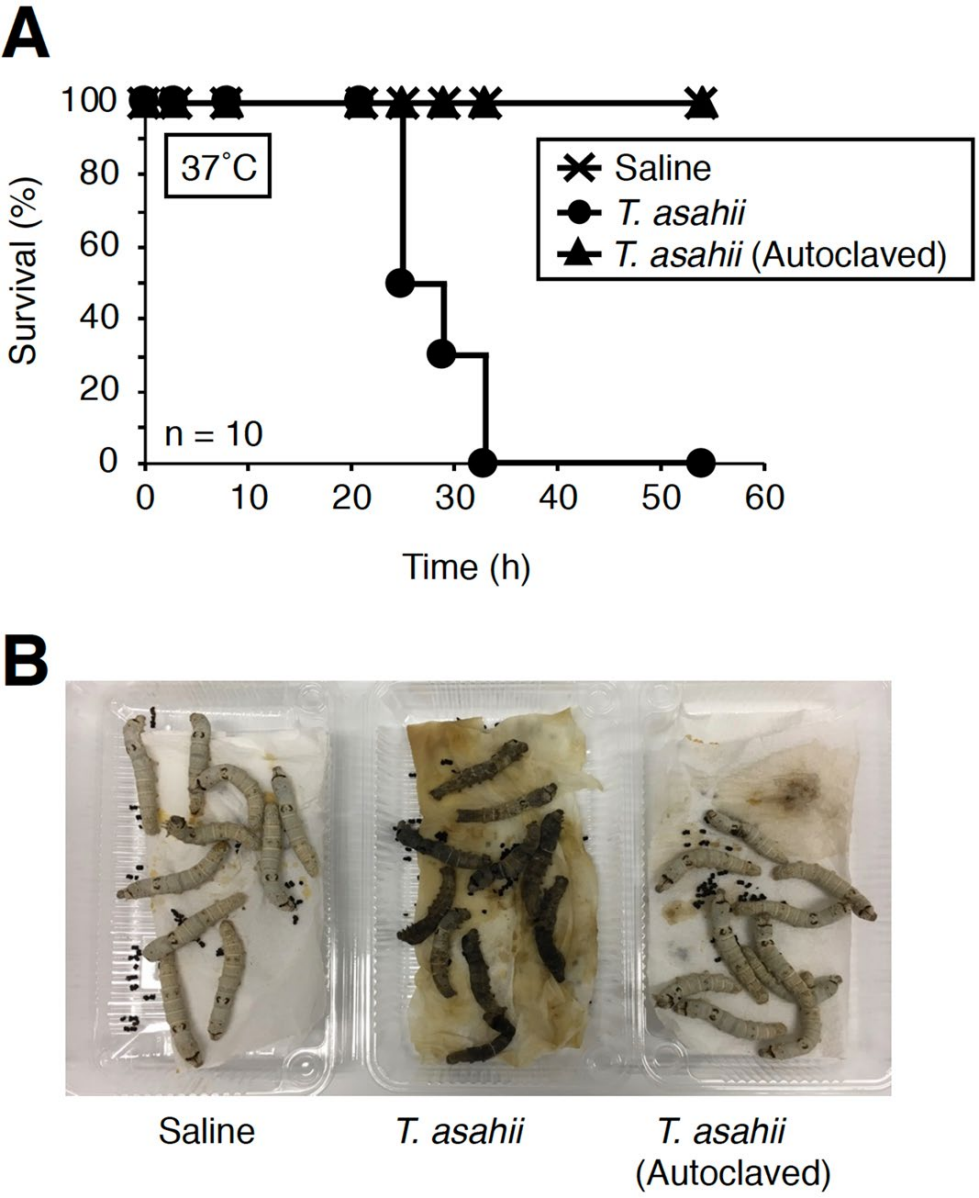
Effect of heat-treatment of *T. asahii* cells on the silkworm killing ability. (**A**) Saline, *T. asahii* JCM2466 (3 × 10^6^ cells), or autoclaved *T. asahii* JCM2466 (autoclaved 3 × 10^6^ cells) was injected into the silkworm hemolymph. Survival of the silkworms at 37 °C was monitored. n = 10/group. (**B**) Silkworms after injection at 48 h under 37 °C incubation are shown.

To establish a silkworm infection model with fungi, the silkworm rearing temperature after infection should be determined^24,48^. In *Cryptococcus neoformans* (H99 strain), by injection of 10^7^ cells into silkworm hemolymph, the silkworms survived for 2 days rearing at 27 °C, but all silkworms died within 2 days at 37 °C^27^. Therefore, we first determined the rearing temperature at which *T. asahii* would kill silkworms. When silkworms injected with 1.9 × 10^6^ cells of *T. asahii* were reared at 27 °C, most of silkworms survived at 48 h, but when reared at 37 °C, all of the silkworms died within 48 h (Fig. 1A–C). Hyphal formation of *T. asahii* was observed in the silkworm hemolymph after *T. asahii* injection under 37 °C rearing conditions (Fig. 1D). Silkworms died in a dose-dependent manner after administering *T. asahii* (Fig. 2A). The LD_50_ was 3.9 × 10^5^ cells (Fig. 2B and Supplementary Fig. 1). On the other hand, silkworms injected with autoclave-treated *T. asahii* cells did not die (Fig. 3A,B). We previously reported the LD_50_ value of *C. neoformans* after 48 h at 37 °C^27^, which are the same experimental conditions we used to determine the LD_50_ value of *T. asahii*. The LD_50_ value of the *C. neoformans* H99 strain was 6 × 10^6^ cells per larva. The LD_50_ value of the *T. asahii* JCM2466 strain determined in this study is lower than that of the *C. neoformans* H99 strain, indicating that the pathogenicity of *T. asahii* JCM2466 strain is higher than that of the *C. neoformans* H99 strain under these experimental conditions.

### Evaluation of the therapeutic effects of antifungal drugs using the silkworm infection model

**Figure 4:**
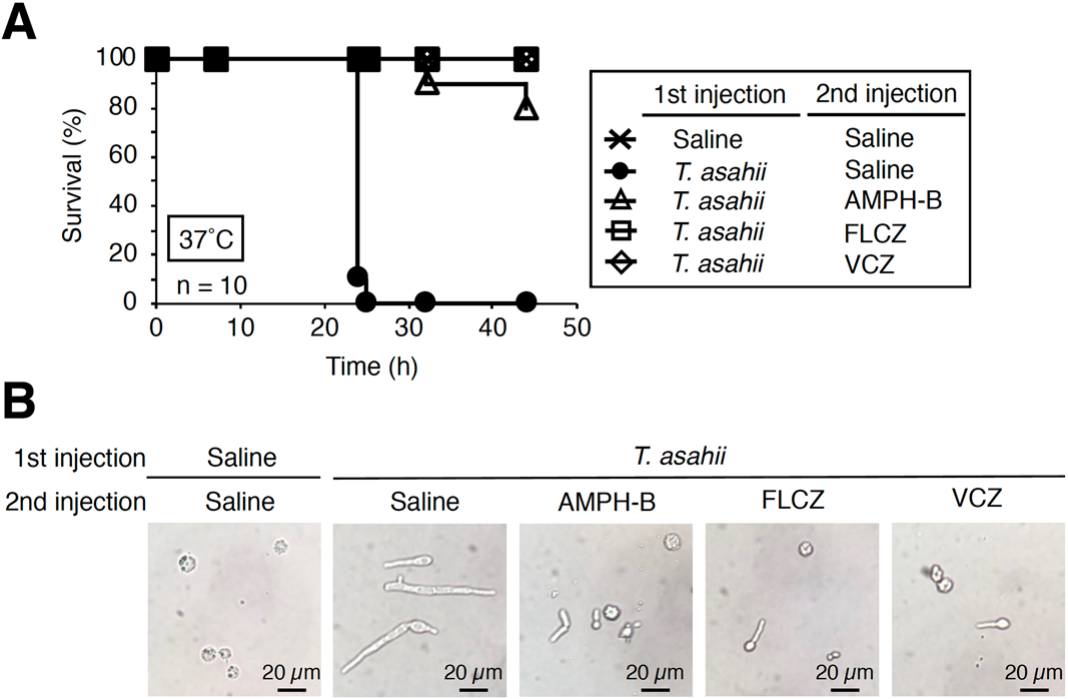
Therapeutic effects of antifungal drugs in silkworms infected with *T. asahii.* (**A**) Saline or *T. asahii* JCM2466 (3 × 10^6^ cells) was injected into the silkworm hemolymph, followed by injection of 50 µl of amphotericin B (AMPH-B, 100 µg/ml), fluconazole (FLCZ, 100 µg/ml), or voriconazole (VCZ, 100 µg/ml) into the hemolymph. Silkworms were reared at 37 °C and survival of the silkworms was monitored. (**B**) Silkworm hemolymph was collected at 24 h after injection and observed with a microscope.

We previously found that the therapeutic effect of antifungal drugs could be evaluated using a silkworm infection model with *C. neoformans*^27^. Administration of the antifungal drugs amphotericin B, fluconazole, and voriconazole prolonged the silkworm survival time after injection of *T. asahii* (Fig. 4A). The ED_50_ value of amphotericin B, fluconazole, and voriconazole was 1.3, 3.9, and 0.4 µg g^−1^ of larva, respectively (Table 2 and Supplementary Fig. 2). Hyphal formation of *T. asahii* in the silkworm hemolymph at 24 h after injection was inhibited by administering amphotericin B, fluconazole, and voriconazole (Fig. 4B). These findings suggest that the therapeutic effect of antifungal drugs can be quantitatively evaluated using the silkworm infection model with *T. asahii*.

**Table 2:** ED_50_ and MIC values of amphotericin B, fluconazole, and voriconazole against *T. asahii* JCM2466.

	ED_50_ (µg g^−1^ of larva)	MIC (µg ml^−1^)
Amphotericin B	1.3	1
Fluconazole	3.9	8
Voriconazole	0.4	0.25

### Establishment of a transgenic *T. asahii* strain expressing eGFP

**Figure 5:**
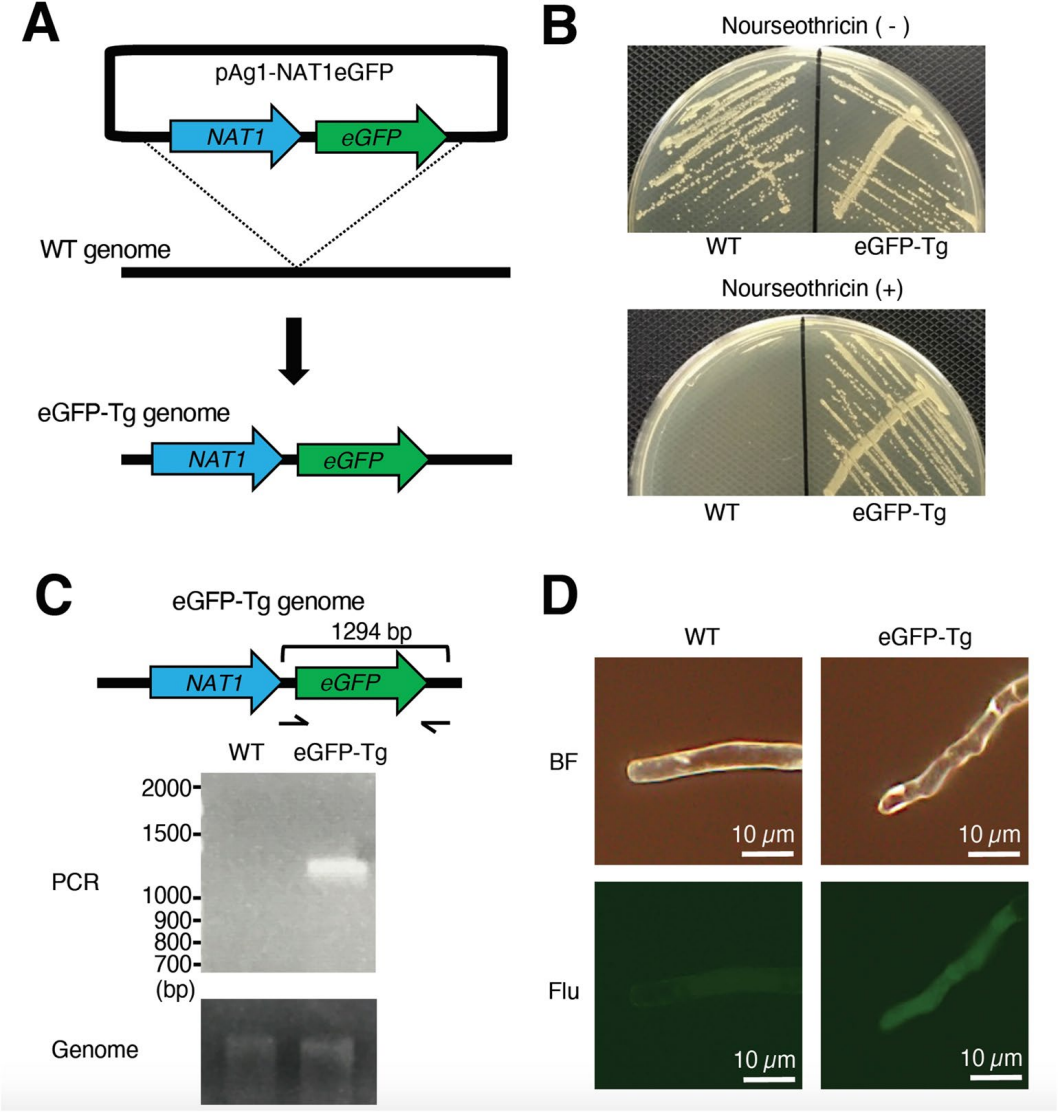
Establishment of transgenic *T. asahii* expressing eGFP. (**A**) Schematic illustration of gene transfer of the nourseothricin resistance gene *NAT1* and the gene encoding eGFP to the *T. asahii* JCM2466 strain. (**B**) JCM2466 strain and JCM2466 eGFP-Tg strains were spread on Sabouraud agar medium containing nourseothricin (50 µg/ml) and incubated at 27 °C for 2 days. (**C**) Detection of gene encoding eGFP in the chromosome of the *T. asahii* strain by PCR. Full-length gels are presented in Supplementary Fig. 5. (**D**) The JCM2466 strain and the *T. asahii* eGFP-Tg strain were observed with a fluorescence microscope. BF: bright field. Flu: fluorescent, which is field of view under conditions of irradiation with excitation light for fluorescence detection.

By gene recombination using *Agrobacterium tumefaciens*, we successfully introduced a gene into dermatophytes, which cause a superficial cutaneous fungal infection^46^. Using the *Agrobacterium* gene transfer method, a plasmid inserted with a nourseothricin resistance gene and a gene encoding eGFP was introduced into *T. asahii* to obtain a strain that grew on agar medium containing nourseothricin (Fig. 5A,B). When the genome of the nourseothricin-resistant strain was used as a template, a band of the expected size of the eGFP gene was amplified by PCR, but when the wild strain genome was used as a template, no amplification was observed (Fig. 5C). Moreover, the nourseothricin-resistant strain emitted green fluorescence when irradiated with excitation light (Fig. 5D). We named the strain eGFP-Tg. These results suggest that a nourseothricin-resistance gene and gene encoding eGFP could be introduced into *T. asahii* by gene recombination using *A. tumefaciens*.

### Fluorescence imaging analysis of *T. asahii* infection

**Figure 6:**
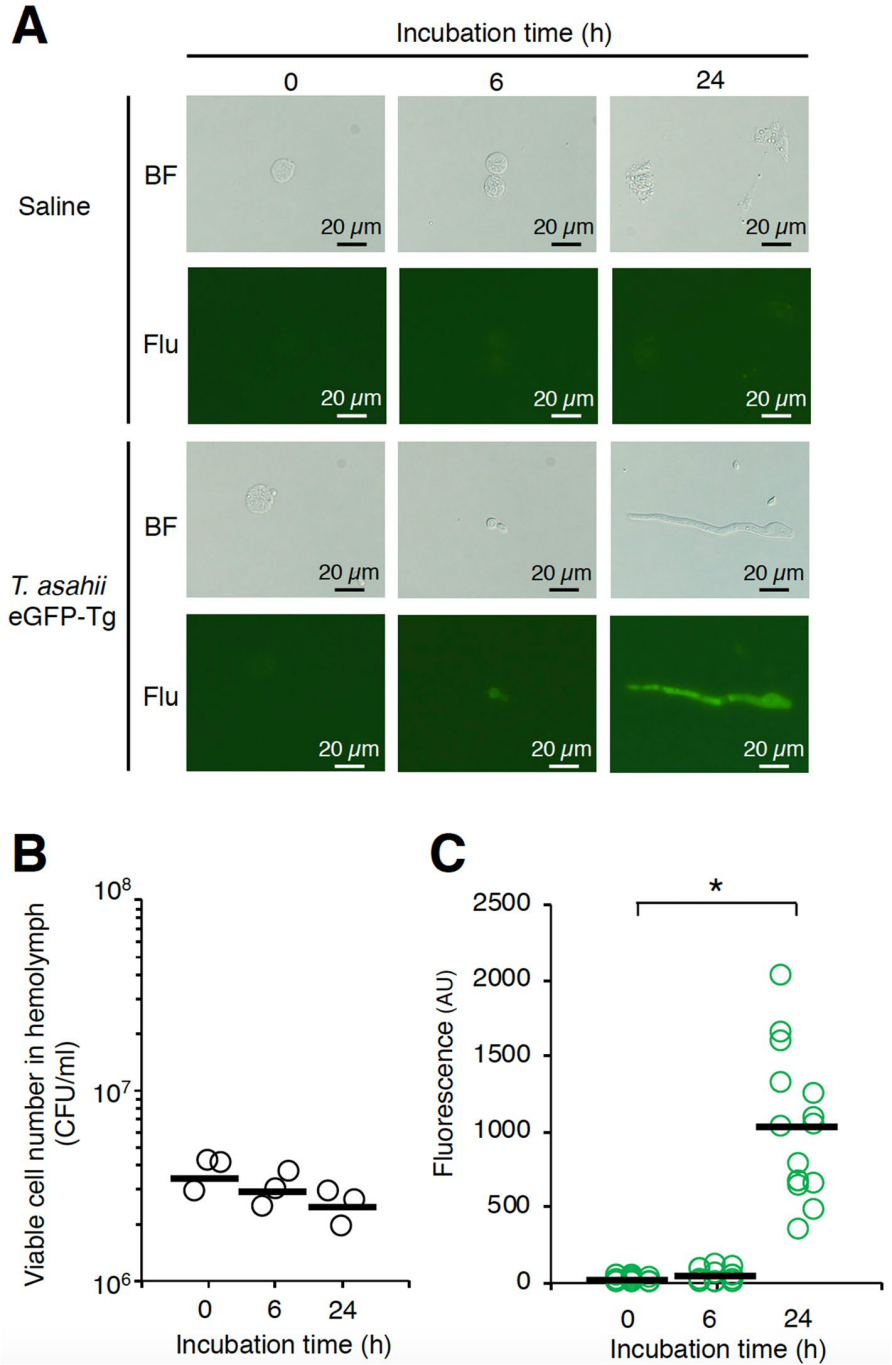
Growth of transgenic *T. asahii* expressing eGFP in silkworm hemolymph. Saline or *T. asahii* JCM2466 eGFP-Tg strain (1 × 10^6^ cells) was injected into the silkworm hemolymph. Silkworm hemolymph was collected at 0, 6, and 24 h after injection and observed with a fluorescence microscope (**A**). The colony forming units (**B**) and fluorescence (**C**) were calculated. BF: bright field. Flu: fluorescent, in which the field of view was irradiated with excitation light for fluorescence detection. Scale bar, 20 µm. Statistically significant differences between groups were evaluated using Tukey–Kramer method. *P < 0.05.

**Figure 7:**
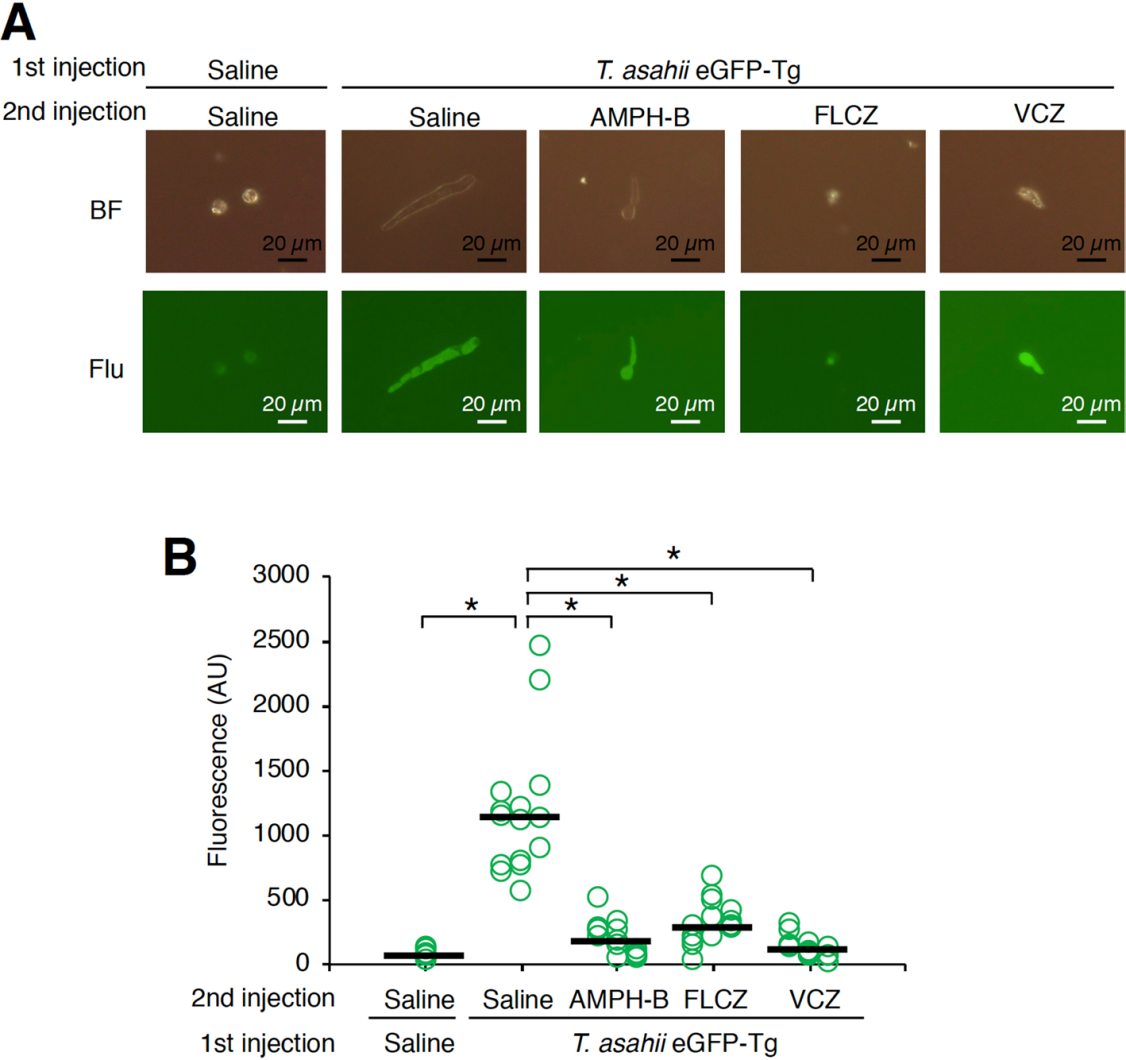
Antifungal drug-induced inhibition of growth of transgenic *T. asahii* expressing eGFP in silkworm hemolymph. (**A**) Saline or *T. asahii* JCM2466 eGFP-Tg strain (3 × 10^6^ cells) was injected into the silkworm hemolymph, followed by injection of 50 µl of amphotericin B (AMPH-B, 100 µg/ml), fluconazole (FLCZ, 100 µg/ml), or voriconazole (VCZ, 100 µg/ml) into the hemolymph. Silkworm hemolymph was collected at 24 h after injection and observed with a fluorescence microscope (**A**). The fluorescence was calculated (**B**). Statistically significant differences between groups were evaluated using Tukey–Kramer method. *P < 0.05.

We examined whether *T. asahii* grew in silkworms by microscopic observation of the hemolymph of silkworms infected with *T. asahii*. Hyphal formation was observed in the silkworm hemolymph at 24 h after injection of the *T. asahii* eGFP-Tg strain (Fig. 6A). On the other hand, we observed no increase in the number of colony-forming units of *T. asahii* in the hemolymph (Fig. 6B). We quantified the hyphal formation of dermatophytes in silkworms by measuring fluorescence intensity using transgenic dermatophytes expressing eGFP^43^. The fluorescence intensity increased in hemolymph of silkworms at 24 h after injection of *T. asahii* eGFP-Tg strain (Fig. 6C). The increased fluorescence intensity was attenuated by administering the antifungal drugs amphotericin B, fluconazole, and voriconazole (Fig. 7A,B). We also confirmed that the pathogenicity of the *T. asahii* eGFP-Tg strain in the silkworm infection model was similar to that of the wild-type strain (Supplementary Figs. 3 and 4). These results suggest that fluorescence imaging using the transgenic *T. asahii* strain expressing eGFP is effective for quantitatively evaluating hyphal formation in the host.

## Discussion

We established a silkworm infection model with *T. asahii* and evaluated the therapeutic effects of antifungal drugs using the silkworm infection model. Moreover, we generated a transgenic *T. asahii* strain expressing eGFP by gene recombination using *A. tumefaciens*. The silkworm infection model and the *T. asahii* gene recombination technology will be useful for elucidating the infection mechanisms of *T. asahii* and developing antifungal drugs.

Silkworm systemic infection models caused by pathogenic fungi, such as *C. albicans*, *C. tropicalis*, *C. glabrata*, *C. neoformans*, *Aspergillus fumigatus*, and dermatophytes, are reported^26,27,29,30,38,43^. *T. asahii* infection models using mice with neutropenia and *Galleria mellonella*, an insect, have been reported^4^. In these infection models, the number of viable *T. asahii* cells in the host organs is measured by colony-forming units and therefore quantifying the actual cell proliferation of *T. asahii* may not be accurate. The fluorescence quantification method using the transgenic *T. asahii* expressing eGFP can be used to quantify cell proliferation by considering hyphal formation in the infection models using mice and *Galleria mellonella*.

We demonstrated that the death of silkworms caused by *T. asahii* was abolished by autoclaving the fungal cells, and that antifungal drugs inhibited the hyphal formation in silkworms and effectively treated silkworm infections. *T. asahii* also forms arthroconidia that are segmented conidia. The arthroconidia of *T. asahii* were observed in multinucleated giant cells present in the blood of a patient with Job’s syndrome^49^. To our knowledge, however, the molecular mechanisms involved in hyphal and arthroconidia formation in *T. asahii* are unknown. Genetic evidence from experiments using a mutant that cannot form hyphae or arthroconidia is needed to clarify the contribution of hyphal and arthroconidia formation to the pathogenicity of *T. asahii*. Silkworms are useful for identifying virulence genes of pathogenic microorganisms^24^. Novel pathogenesis-related genes of *Staphylococcus aureus*, *Pseudomonas aeruginosa*, *Bacillus cereus*, *C. albicans*, and *C. glabrata* have been identified by screening mutants with reduced pathogenicity in silkworms from genetic-disrupted mutant libraries^19,29,30,50,51^. We previously reported in generating gene-deficient strains of dermatophytes by gene recombination using *A. tumefaciens*^46^. In the present study, we revealed that gene recombination using *A. tumefaciens* is a useful transgenic technique in *T. asahii*. Future studies will establish gene-deficient strains and screening for virulence genes in *T. asahii*. On the other hand, in the experimental system established in this study, multiple experiments are required to determine LD_50_ value by the simple logistic regression model. Moreover, there are the difference in LD_50_ values between each experiment (Supplementary Fig. 1). Optimizing the experimental condition to enable more reproducible measurement of LD_50_ values is a future subject.

Silkworms are useful for evaluating the pharmacokinetics and toxicity of compounds^31–35^. In mammalian animals, organs such as the intestinal tract, liver, and kidney govern the pharmacokinetics of drugs. Recent studies revealed that silkworms have functionally similar organs that affect drug pharmacokinetics and toxicity^32–35^. Many in vitro and in vivo analyses revealed that the absorption of compounds from the silkworm intestinal tract is similar to that in mammals^26,27,31,43^.

The ED_50_ values of antibiotics in silkworms are similar to those in mice^26^. The difference in the total clearance, volume of distribution, and half-life values of anti-microbial agents such as chloramphenicol, tetracycline, vancomycin, rifampicin, micafungin, and fluconazole is less than 10-fold between silkworms and mammals^35^. The LD_50_ values for compounds are also similar between silkworms and mammals^28,32^. Lysocin E, nosokomycin, ASP2397, and GPI0363 were identified by exploratory research using a silkworm infection model from microbial culture broths and chemical libraries as novel antimicrobial compounds that show therapeutic effects in mouse infection experiments^34,36–38^. Furthermore, we demonstrated that substances identified by in vivo screening using a silkworm disease model are effective in humans^52^. Therefore, silkworms are useful for in vivo screening of compounds that are candidate antibacterial and antifungal agents.

The ED_50_ values of amphotericin B and voriconazole in the silkworm infection model with *T. asahii* were 1.3 mg/kg and 0.4 mg/kg, respectively. A previous study using a guinea pig infection model with *T. asahii* reported amphotericin B and voriconazole ED_50_ values greater than 1.5 mg/kg and 5–10 mg/kg, respectively^53^. Therefore, the ED_50_ value of voriconazole in the silkworm model is lower than that in the guinea pig model. In the previous report using the guinea pig model, antifungal drugs were administered 24 h after infection. On the other hand, in the silkworm infection model, antifungal drugs were administered immediately after infection. The difference in the timing of the administration may account for the difference in ED_50_ values. *T. asahii* exhibits natural resistance to candin antifungals such as micafungin, and strains resistant to azole antifungals such as fluconazole and amphotericin B have also been clinically isolated^12–14^. New antifungal drugs might be obtained by in vivo screening using a silkworm infection model with a multidrug-resistant *T. asahii* strain.

In conclusion, the silkworm *T. asahii* infection model is useful for quantitatively evaluating the pathogenicity of *T. asahii* and the therapeutic effects of antifungal drugs. We also revealed that *T. asahii* can be genetically modified using *Agrobacterium*. The transgenic *T. asahii* expressing eGFP might be useful for in vivo fluorescence imaging in various infection models.

## Supplementary information

Supplementary file1
