# Supplementary material for: A novel silkworm infection model with fluorescence imaging using transgenic Trichosporon asahii expressing eGFP
Source: Sci Rep. 2020 Jul 3;10:10991. doi: 10.1038/s41598-020-67841-6 (PMC7335072; doi:10.1038/s41598-020-67841-6)
Supplement: Supplementary file 1 — Supplementary file1 [file 41598_2020_67841_MOESM1_ESM.pdf]

Supplementary information

A novel silkworm infection model with fluorescence imaging using transgenic *Trichosporon asahii* expressing eGFP

Yasuhiko Matsumoto[1], Hideki Yamazaki[1], Yusuke Yamasaki[1], Yuki Tateyama[1], Tsuyoshi Yamada[2], Takashi Sugita[1]

[1]Department of Microbiology, Meiji Pharmaceutical University, 2-522-1, Noshio, Kiyose, Tokyo 204-8588, Japan.

[2]Teikyo University Institute of Medical Mycology, 359 Otsuka, Hachioji, Tokyo 192-0395, Japan.

*Address correspondence to: Dr. Yasuhiko Matsumoto, Department of Microbiology, Meiji Pharmaceutical University, 2-522-1, Noshio, Kiyose, Tokyo 204-8588, Japan, Tel: +81-42-495-8745, e-mail: ymatsumoto@my-pharm.ac.jp.

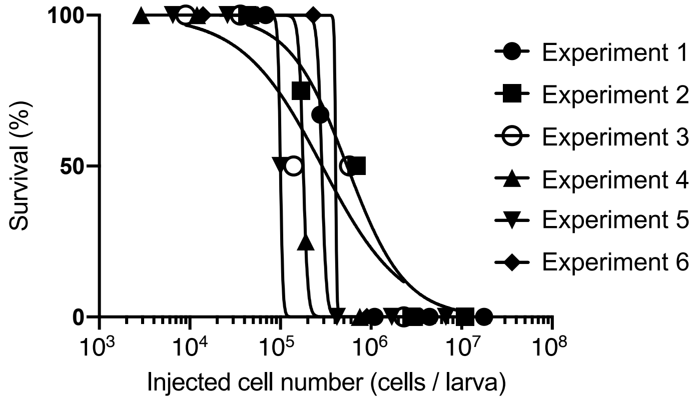

Supplementary Figure 1. Killing of silkworms by *T. asahii* in a dose dependent manner

Survival of the silkworms at 48 h was monitored. Six independent experiments were performed and plotted by non-linear regression using Prism 8 (GraphPad Software, LLC, San Diego, CA, USA, https://www.graphpad.com/scientific-software/prism/).

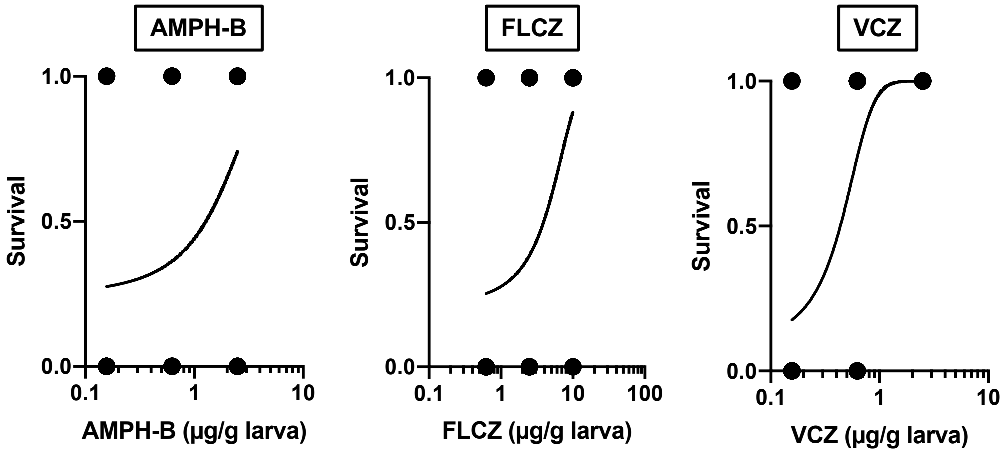

Supplementary Figure 2. Determination of ED_{50} values of antifungal drugs

T. asahii cells (1-5 x 10^6 cells) were injected into the silkworm hemolymph, and various concentrations of the antifungal agents (50 µl) dissolved in saline were injected immediately afterwards into the silkworm hemolymph. The doses were created by 4-fold serial dilutions. To determine the ED_{50} values, 5 or 6 silkworms were injected with each dose of the antifungal agents. Survival of the silkworms at 48 h was monitored. The ED_{50} values were calculated from combined data of 4-5 independent experiments by simple logistic regression model using Prism 8 (GraphPad Software, LLC, San Diego, CA, USA).

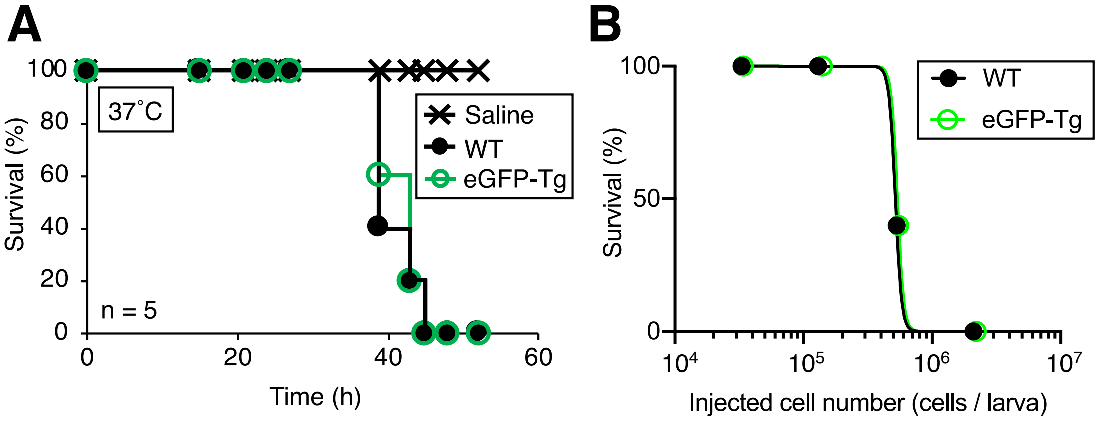

Supplementary Figure 3. Killing of silkworms by injection of *T. asahii* WT and transgenic *T. asahii* expressing eGFP

(**A**) Saline, *T. asahii* WT cells (2.1 x 10^6 cells), or *T. asahii* JCM2466 eGFP-Tg strain (2.2 x 10^6 cells) were injected into the silkworm hemolymph. Survival of the silkworms at 37°C was monitored. n = 5/group. (**B**) Survival of the silkworms at 48 h was monitored and plotted by non-linear regression using Prism 8 (GraphPad Software, LLC, San Diego, CA, USA, https://www.graphpad.com/scientific-software/prism/).

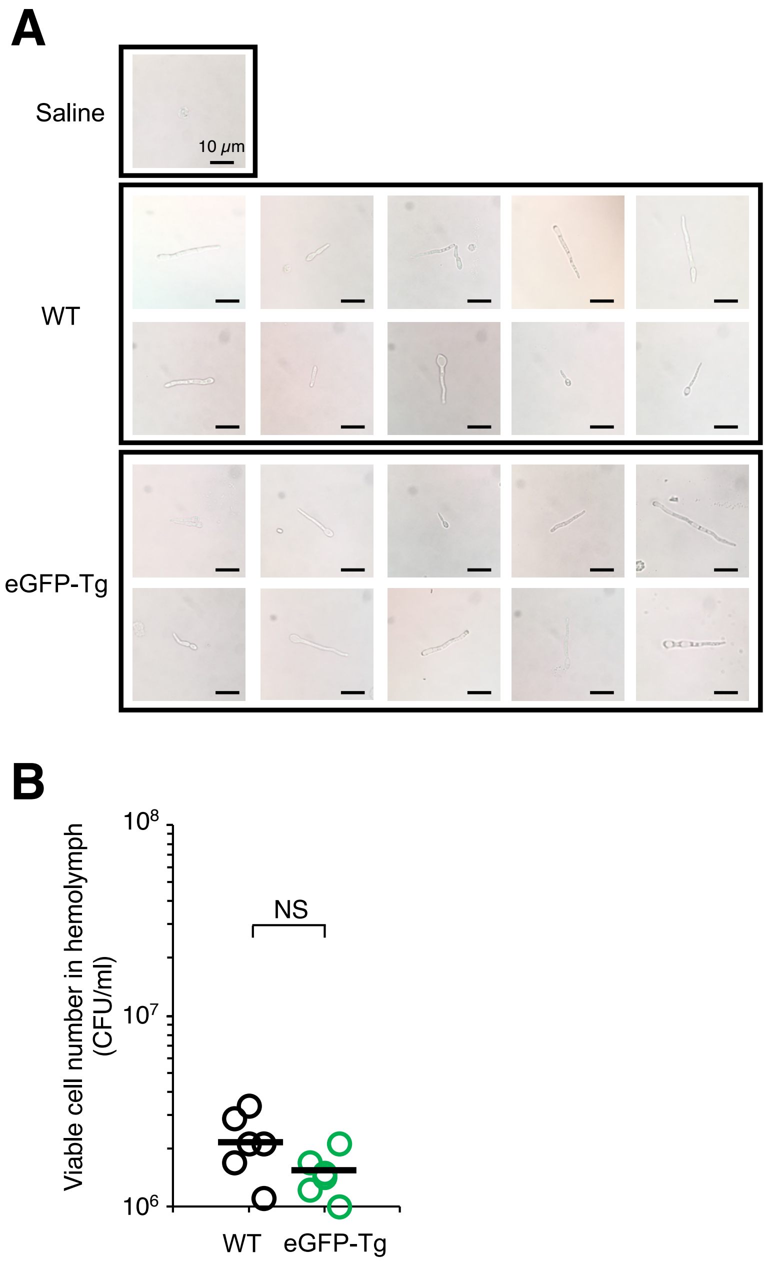

Supplementary Figure 4. Morphology and viable cell number of *T. asahii* WT and transgenic *T. asahii* expressing eGFP in silkworm hemolymph

Saline, *T. asahii* WT cells (2.1 x 10^6 cells), or *T. asahii* JCM2466 eGFP-Tg strain (2.2 x 10^6 cells) were injected into the silkworm hemolymph. Silkworm hemolymph was collected at 24 h after injection and observed with a microscope (**A**). Scale bar, 10 μm. The colony-forming units (**B**) were calculated. Statistically significant differences between groups were evaluated using Student *t*-test. NS: not significant, $P > 0.05$.

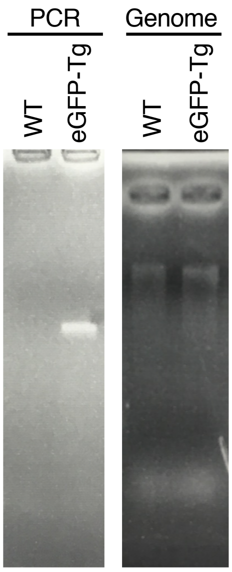

Supplementary Figure 5. Full-length gels of Figure 5C.
